# Community-based culinary and nutrition education intervention promotes fruit and vegetable consumption

**DOI:** 10.1017/S1368980021003797

**Published:** 2022-02

**Authors:** Jessica Jarick Metcalfe, Melissa Pflugh Prescott, Melissa Schumacher, Caitlin Kownacki, Jennifer McCaffrey

**Affiliations:** 1 Department of Food Science and Human Nutrition, University of Illinois at Urbana-Champaign, 905 South Goodwin Avenue, Urbana, IL 61801, USA; 2 Office of Extension and Outreach, University of Illinois at Urbana-Champaign, Urbana, IL, USA

**Keywords:** Cluster randomised controlled trial, Mixed methods, Diet, Family, Community-based intervention

## Abstract

**Objective:**

The main objective of this study was to evaluate the impact of the Market to MyPlate (M2MP) program on fruit and vegetable consumption and cooking behaviours. Secondary objectives were to examine factors that affected participant retention and program completion, and analyse program feedback provided by participants.

**Design:**

This study conducted a mixed methods evaluation embedded within a cluster randomised controlled trial of the M2MP intervention. Adult participants completed a pre- and post-program survey reporting on their fruit and vegetable consumption and cooking behaviours. A subsample participated in structured interviews, providing feedback about M2MP and the impact of the program.

**Setting:**

Seven weekly classes took place in community centres and extension offices in central Illinois.

**Participants:**

120 adults and their families participated. Class cohorts were randomly assigned to one of three treatment groups: (1) nutrition education and cooking classes with produce allocations (PAE, *n* 39); (2) nutrition education and cooking classes only (EO, *n* 36) or (3) control group (*n* 45).

**Results:**

Compared to control, PAE participants reported larger increases from pre- to post-intervention in fruit (*P* = 0·001) and vegetable consumption (*P* = 0·002), with no differences in cooking frequency. Interview analyses identified key themes in behaviour changes due to M2MP, including reported increases in dietary variety, cooking self-efficacy and children’s participation in cooking.

**Conclusions:**

PAE participants who received an intervention that directly increased their access to fresh produce (via produce allocations) increased their reported fruit and vegetable consumption. Though participants’ cooking frequency did not change, interviewees reported increased variety, cooking confidence and family participation in cooking.

Unhealthy dietary intake is recognised as a major public health concern internationally, and low consumption of fruits and vegetables is a key risk factor for a variety of chronic diseases (including diabetes, cancer, obesity and CVD) worldwide^(1–3)^. In the USA, the majority of adults and children do not meet dietary recommendations for consumption of fruits and vegetables^(4–6)^, and research indicates that individuals from low-income families are at even higher risk for unhealthy dietary intake^(7)^, obesity^(8,9)^ and other poor diet-related health outcomes^(10,11)^.

Low-income families often encounter unique obstacles that hinder healthy eating behaviours. Research shows that healthy foods (e.g. fruits and vegetables) are more expensive (per calorie) than less healthy alternatives (e.g. highly processed foods)^(12)^. Purchasing an adequate quantity of healthy foods to consume a well-balanced diet is a significant challenge for families with lower incomes^(13)^, and individuals with more costly diets are more likely to meet the recommendations outlined in the *Dietary Guidelines for Americans* ^(14)^. Families with lower incomes also encounter challenges related to preparation of healthy foods. Less flexible work schedules and barriers related to time make cooking at home more challenging^(15–17)^, and families with lower incomes often face challenges with cooking skills, resources and equipment^(18,19)^. Consequently, interventions geared toward low-income participants can experience issues with recruitment and retention, as potential participants are more likely to experience challenges (e.g. transportation issues, inflexible work hours) that make consistent participation more difficult^(20,21)^. Research that investigates how to best design interventions for vulnerable populations is critical in addressing these important health behaviours. Concerns about the negative impact that unhealthy diets can have on overall health and wellbeing has led experts to advocate for interventions targeting improvements in dietary health with low resource populations.

To address the complex issues related to preparing and consuming nutrient dense foods, effective interventions should address multiple factors facing individuals. Frameworks, such as the social ecological model, that address not only individual factors, but also influences of socio-cultural and environmental factors are recognised as more effective in supporting behavioural changes. These approaches attend to the broader systems and context that participants are situated within, an approach that is particularly important for low resource populations who may be more likely to experience challenges in accessing healthy foods^(22)^. Nutrition interventions targeting increased access to healthy foods have the potential to improve dietary behaviours and reduce disparities experienced by low resource populations^(23)^. The Market to MyPlate (M2MP) community-based intervention program was developed to provide family nutrition education and hands-on cooking classes for low-income individuals and their families and increase participants’ access to healthy foods. The objective of this study was to evaluate the impact of the M2MP program on participants’ reported fruit and vegetable consumption and cooking behaviours. A secondary aim was to better understand factors that influence retention or participants’ completion of the M2MP program and analyse program feedback from participants.

## Methods

### Study design and setting

This study used an embedded mixed methods design in which the quantitative survey data and qualitative interview data were analysed within the framework of a cluster randomised controlled trial^(24,25)^. The M2MP program consisted of a 7-week in-person nutrition education and hands-on cooking intervention in which class cohorts were randomised to one of three conditions: (1) produce allocations with educational classes (PAE); (2) educational classes only (EO); or (3) control group (which received a delayed PAE intervention). The control group participants received no intervention or education between their completion of the pre-intervention and post-intervention surveys, but received a delayed PAE intervention after completing the post-intervention surveys. The M2MP program was delivered in partnership with the Expanded Food & Nutrition Education Program and Supplemental Nutrition Assistance Program-Education programs operated by University of Illinois Extension, and classes were taught by Expanded Food & Nutrition Education Program community workers who were trained to deliver nutrition education. Expanded Food & Nutrition Education Program and Supplemental Nutrition Assistance Program-Education are nutrition education and obesity prevention programs for low-income populations in the USA whose goal is to improve the likelihood that persons eligible for SNAP will make nutritious food choices within a limited budget and choose physically active lifestyles. M2MP classes took place in central Illinois at a local Extension Office or community centres with kitchen facilities.

### Participants

Participants were recruited in April–September of 2018 from five community sites that serve low-income populations including local food pantries and WIC (Supplemental Nutrition Program for Women, Infants and Children) offices. Participants (*n* 2) were excluded if they had already participated in an Extension nutrition education program in the past year. Eligibility criteria were the same for all conditions (PAE, EO and control), and required participants to be primarily responsible for or actively involved in preparing meals for their family. Researchers randomly assigned each of the sixteen class cohorts (i.e. a weekly class meeting time for a period of 7 weeks) to one of the three conditions via block randomisation to ensure balanced distribution of cohorts across conditions. Participants self-selected class cohorts that worked with their schedules and availability. Participants were not informed of their condition assignments. When participants selected a cohort that was assigned as a control, they were informed of the delayed control design and were signed up to receive the PAE intervention in the subsequent 7-week period. Quantitative data was collected from the full sample of participants via pre- and post-program Food and Physical Activity Questionnaires^(26)^. Qualitative data was collected from a subsample of participants (spread across the two treatment conditions) who had volunteered to participate in interviews to provide feedback about the M2MP program. To participate in interviews, participants were required to attend at least six of the seven M2MP classes (all eligible participants were invited to volunteer). This study was approved by the Institutional Review Board of the University of Illinois at Urbana-Champaign (Protocol # 17 806), and all participants gave written informed consent.

### Sampling

The sample size was limited by the number of classes/cohorts, which were determined by the availability of Extension staff to teach M2MP classes. Since this was an exploratory study, power and sample size calculations were not conducted (as the intent was to explore potential outcomes of interest via exploratory hypotheses, not to achieve statistical significance on a specific outcome).

### Intervention

M2MP is a 7-week nutrition education and hands-on cooking intervention (with 2 h lessons) for low-income adults and their families. Participants were required to complete at least five of the seven total M2MP classes to receive post-intervention incentives, but all participants who were present at the last class were invited to participate in post-intervention data collection (regardless of attendance rate). The program utilised an evidence-based nutrition and cooking education curriculum^(27)^ supplemented with education about how to use food assistance (SNAP/Women, Infants and Children) benefits at local farmers’ markets. PAE and EO participants received the same educational intervention and followed the same curriculum. PAE participants were given an assortment of fresh produce (mostly vegetables and some herbs) from a local farm to take home after each of the seven classes, while EO participants received produce coupons (of equal value) after the conclusion of the intervention that could be redeemed at the farmers’ market or a local farm.

The M2MP intervention was based on the social ecological framework, which took into consideration aspects of improving dietary quality based on individual factors, social influences, the community and the environmental setting. At the individual level, participants engaged in hands-on culinary classes based on the dietary recommendations of MyPlate. Topics included shopping, cooking and consuming healthful foods, especially fresh produce. As part of the classes, social aspects were reinforced by allowing adults to participate with their children (childcare was provided for children who were too young to participate in classes). Additional social reinforcement was provided through the distribution of recipe books (which included both recipes cooked in class and additional recipes) at the conclusion of the 7-week program to encourage them to use M2MP recipes with their family at home. Aspects of the community and environment included targeted education about where to shop for local sources of produce and how to utilise community food assistance program (SNAP and Women, Infants and Children) benefits at farmers’ markets.

### Data collection

#### Survey data

Participants completed a valid and reliable pre- and post-program Food and Physical Activity Questionnaire^(26)^ at the beginning of the first class and end of the last class. Self-reported demographic information included participants’ age, gender, race, ethnicity, number of children, monthly food budget and nutrition assistance program participation (SNAP or Women, Infants and Children). Five items were selected *a priori* from the twenty question Food and Physical Activity Questionnaire and identified as outcomes of interest in this study (other items, e.g. physical activity behaviours, were not the target of this intervention and therefore are not analysed in this study). Each outcome of interest (cooking frequency, consumption of fruit, vegetables, red or orange vegetables and dark green vegetables) was measured with a single item from the survey. All survey questions were measured on a scale from 1 to 6 points (1 = minimum score, 6 = maximum score for each outcome), with higher scores indicating a higher frequency of the behaviour in question. Fruit and vegetable consumption (‘How many times a day do you eat fruit/vegetables?’) were measured on the following scale: 1 = I rarely eat fruit/vegetables, 2 = Less than 1 time a day (a couple times a week), 3 = 1 time a day, 4 = 2 times a day, 5 = 3 times a day, 6 = 4 or more times a day. Consumption of red or orange and dark green vegetables (‘Over the last week, how many days did you eat red or orange vegetables/dark green vegetables?’) were measured on the following scale: 1 = I did not eat red or orange/dark green vegetables, 2 = 1 day a week, 3 = 2 days a week, 4 = 3 days a week, 5 = 4 or 5 days a week, 6 = 6 or 7 days a week. Cooking frequency (‘How many days a week do you cook dinner (your main meal) at home?’) was measured on the following scale: 1 = I rarely cook dinner at home, 2 = 1 day a week, 3 = 2 days a week, 4 = 3 days a week, 5 = 4 or 5 days a week, 6 = 6 or 7 a week. Survey data were dual-entered by two trained research assistants using a standardised electronic form. The first author then compared their entered data for accuracy and reconciled any discrepancies.

#### Interview data

Structured interviews were conducted with a subsample of participants from across the two treatment conditions to collect program feedback. A structured interview protocol was used to guide discussion, which prompted participants to share their feedback about the program, and asked about how participating in M2MP impacted their eating and cooking behaviours. Interviews lasted from 25 to 50 min, and were audiotaped and transcribed verbatim. Transcripts and interview recordings were reviewed by trained research assistants to ensure content accuracy.

### Data analysis

#### Survey data

Differences between conditions in demographic variables were assessed using ANOVA for quantitative variables and *χ* ^2^ analyses for categorical variables. Before addressing missing outcome data, researchers analysed demographic differences between participants who did (*n* 80) and did not (*n* 40) complete the M2MP program. Differences between those who did and did not complete the program were analysed using *t*-tests for quantitative variables and *χ* ^2^ analyses for categorical variables. Descriptive and program completion analyses were performed using SPSS software, version 24^(28)^.

Multiple imputation methods were used to impute missing data for participants (*n* 40, 33 % of sample) who did not complete the post-survey or were missing any other data. Modern imputation methods such as multiple imputation produce less biased estimates than more traditional methods like mean substitution or list-wise deletion of participants with incomplete data, and may be used when a substantial proportion of the data are missing^(29)^. Using multiple imputation to address incomplete survey data follows best practices for interventions with high attrition rates^(30)^, and were appropriate in this study given that approximately 33 % of participants did not complete the post-program survey. Missing data were imputed in ten datasets using the Fully Conditional Specification method, and pooled estimates (aggregated imputed values) were used for all outcome analyses.

Multilevel modelling was used for outcome analyses to account for the clustering of participants within M2MP class cohorts. Multilevel linear regression analyses were used to assess differences between conditions in pre- to post-program changes in survey scores. All outcomes were measured at the individual level. These regressions controlled for the following class-level variables: seasonality (month when post-program data were collected) and program location (community centre *v*. Extension office), and the following individual-level variables: age, gender, race, ethnicity, number of children, M2MP program completion, distance between home and M2MP program location, monthly food budget and food assistance program participation. All multilevel modelling outcome analyses were performed using HLM software, version 8^(31)^. This study was exploratory, and as such used exploratory outcome analyses that were intended to guide the development of future confirmatory hypotheses.

#### Interview data

Qualitative interview data were dual-coded and analysed using ATLAS.ti software (version 8). Researchers employed a hybrid deductive–inductive methodology, in which the research questions informed the development of the initial codebook and additional unique themes were added as they emerged during the coding process^(32)^. Each transcript was coded by two members of the research team, and discrepancies in codes were discussed and reconciled based on consensus between the two coders. Data were assessed using thematic analysis to identify common themes based on the frequency and intensity of participant comments^(33)^. Quantitative variables (binary: yes/no) were created based on the presence of interview codes to calculate descriptive frequencies quantifying how many participants reported a specific behaviour (e.g. using M2MP recipes at home).

## Results

**Fig. 1:**
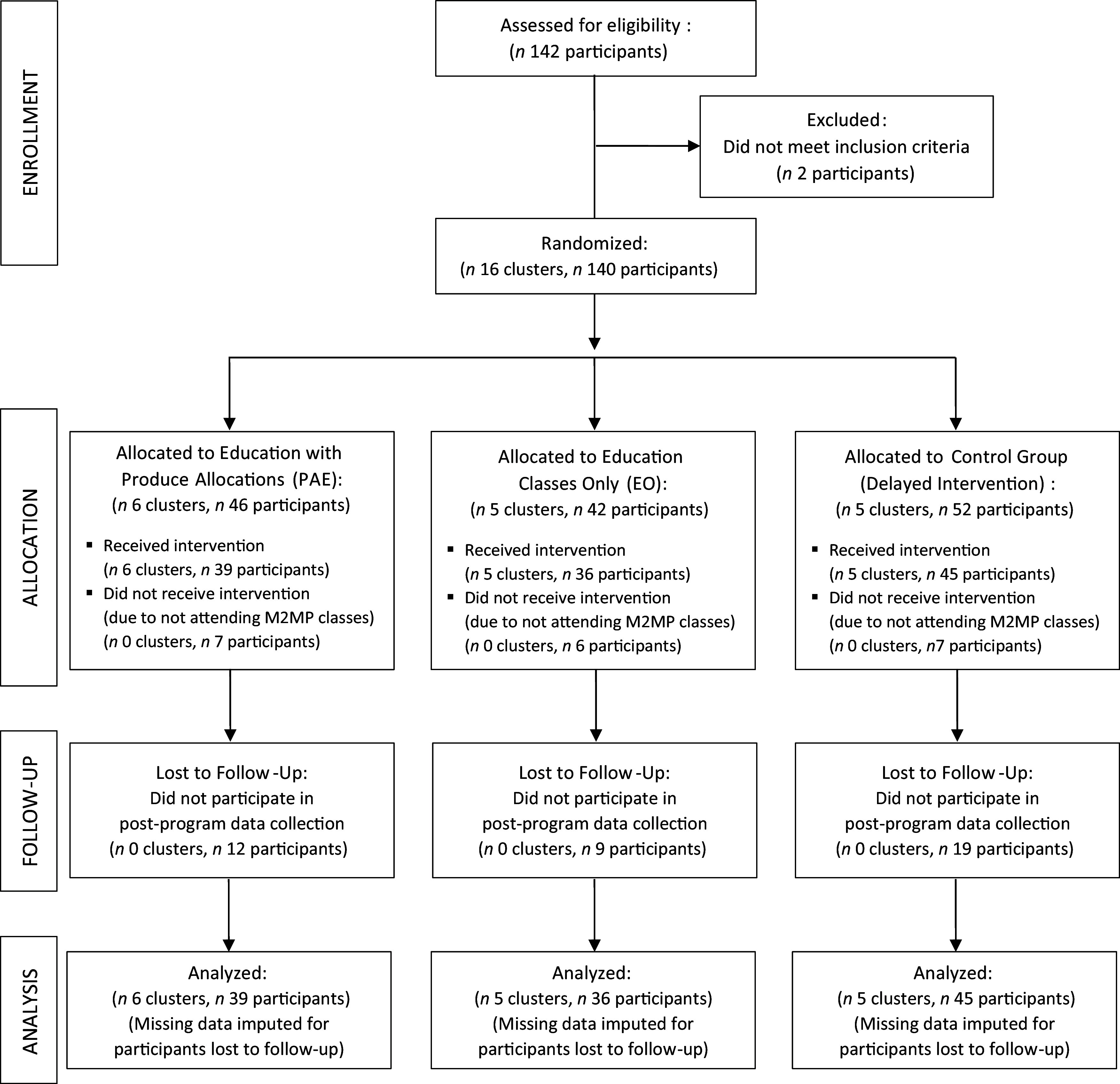
Study flow diagram in accordance with the CONSORT statement for cluster trials

Figure 1 shows a CONSORT study flow diagram describing the number of clusters (class cohorts) and individual participants at each phase of the trial^(34)^. A total of sixteen class cohorts (and 140 individual participants) were randomised to the PAE (*n* 6 clusters, *n* 46 participants), EO (*n* 5 clusters, *n* 42 participants) and control (*n* 5 clusters, *n* 52 participants) conditions. Twenty participants across conditions did not receive the intervention or participate in data collection because they did not attend any M2MP classes. Forty individual participants were lost to follow-up, but data were imputed for these participants, resulting in a total of 120 participants in the analytic sample across the education with PAE (*n* 39 participants), EO (*n* 36 participants) and control conditions (*n* 45 participants).

Descriptive statistics can be found in Table 1. Participants in the PAE condition reported having larger monthly food budgets than participants in the control group. On average, participants in the PAE condition had more children than participants in the education only and control conditions. There were more females than males in the sample (and in each condition), but the gender distribution was not different across conditions. Participants’ average age did not differ significantly across conditions and ranged from 36 to 42 years old. The sample had a relatively diverse racial and ethnic makeup that did not differ significantly between conditions.

**Table 1:** Demographic characteristics of the M2MP sample as a whole and by condition

	Full sample (*n* 120)		Produce and education (*n* 39)		Education only (*n* 36)		Control group (*n* 45)		*P*
	%	*n*	%	*n*	%	*n*	%	*n*	
Age									0·076
Mean	40		36		40		42		
sd	12		10		11		14		
Gender									0·071
Female	72 %	86	82 %	32	75 %	27	60 %	27	
Male	28 %	34	18 %	7	25 %	9	40 %	18	
Race									0·128
Asian	14 %	17	13 %	5	25 %	9	7 %	3	
Black	34 %	41	26 %	10	33 %	12	42 %	19	
Multiracial	18 %	21	20 %	8	9 %	3	22 %	10	
White	34 %	41	41 %	16	33 %	12	29 %	13	
Hispanic/Latino	13 %	15	22 %	8	8 %	3	9 %	4	0·183
SNAP or WIC Participant	47 %	56	59 %	23	33 %	12	47 %	21	0·084
Monthly food budget									0·006
Mean	$344		$407		$350		$285		
sd	176		202		147		156		
Number of children									0·008
Mean	1·6		2·2		1·3		1·4		
sd	1·3		1·4		1·2		1·2		
Completed M2MP program	67 %	80	69 %	27	75 %	27	58 %	26	0·242

*Note.* Differences in demographics between conditions were analysed using ANOVA for quantitative variables and *χ* ^2^ analyses for categorical variables. M2MP, Market to MyPlate. SNAP, Supplemental Nutrition Assistance Program.

### Survey results

#### Program completion

Compared to those who completed the post-program survey (completers), those who did not provide follow-up data (non-completers) had fewer children, smaller food budgets, were less likely to be Asian and more likely to be African American and lived farther from the M2MP site where they attended program classes (see Table 2). Completers and non-completers did not differ significantly in age, gender distribution, ethnicity, food assistance program participation, monthly food budget or class location (community centre *v*. Extension office).

**Table 2:** Demographic characteristics of participants who did and did not complete the M2MP program

	Completed program (*n* 80)		Did not complete program (*n* 40)		*P*
	%	*n*	%	*n*	
Age					0·217
Mean	39		42		
sd	11		14		
Gender					0·522
Female	74 %	59	68 %	27	
Male	26 %	21	32 %	13	
Race					0·009
Asian	21 %	17	0 %		
Black	28 %	22	48 %	19	
Multiracial	18 %	14	18 %	7	
White	34 %	27	35 %	14	
Hispanic/Latino	13 %	10	13 %	5	0·606
SNAP or WIC participant	45 %	36	50 %	20	0·373
Monthly food budget					0·023
Mean	$370		$293		
sd	173		174		
Number of children					0·046
Mean	1·8		1·3		
sd	1·3		1·3		
Participated at community centre	38 %	30	23 %	9	0·072
Lived < 2 miles from M2MP site	39 %	31	20 %	8	0·029

*Note.* Differences between participants who did and did not complete the program were analysed using *t*-tests for quantitative variables and *χ* ^2^ analyses for categorical variables. M2MP, Market to MyPlate; WIC, Women, Infants and Children.

#### Program impact

Multilevel modelling analyses comparing pre- to post-program changes in survey scores between conditions are presented in Table 3. Compared to control group participants, participants in the PAE group reported larger increases (from pre- to post-intervention) in consumption of fruits (1·31 points larger increase than control, *P* = 0·001) and vegetables (1·04 points larger increase than control, *P* = 0·002). There were no significant differences between PAE and control participants in red and orange vegetable consumption, dark green vegetable consumption or cooking frequency. There were no significant differences between the control group and the EO group in any program outcomes.

**Table 3:** Multilevel linear model (MLM) analyses for differences between treatment and control groups in pre- to post-program change scores for frequency of eating fruits and vegetables and cooking main meal at home (*n* 120)

	Control group				Education only							Education & produce allocations						
	Pre-test mean	95 % CI	Pre-post mean change	95 % CI	Pre-test mean	95 % CI	Pre–post mean change	95 % CI	*β*	se	*P*	Pre-test mean	95 % CI	Pre-post change mean	95 % CI	*β*	se	*P*
Eat fruit	3·70	3·22, 4·18	−0·31	−0·73, 0·11	3·20	2·83, 3·57	−0·18	−0·80, 0·62	0·13	0·28	0·690	2·36	1·83, 2·89	+1·00	0·45, 1·55	1·31	0·38	0·001
Eat vegetables	3·99	3·38, 4·60	−0·37	−0·82, 0·08	3·65	3·19, 4·11	−0·25	−0·62, 0·12	0·08	0·27	0·778	3·40	2·91, 3·89	+0·67	0·26, 1·08	1·04	0·32	0·002
Eat dark green vegetables	3·68	3·03, 4·33	−0·16	−0·61, 0·29	3·58	3·09, 4·07	+0·13	−0·34, 0·60	0·29	0·47	0·262	3·16	2·73, 3·59	+0·57	0·03, 1·11	0·73	0·45	0·108
Eat red/orange vegetables	3·52	2·91, 4·13	−0·24	−0·82, 0·34	3·34	2·79, 3·89	+0·50	0·16, 0·84	0·74	0·41	0·081	3·08	2·67, 3·49	+0·48	0·09, 0·87	0·72	0·42	0·091
Cook dinner/main meal at home	4·63	4·21, 5·05	−0·26	−0·54, 0·28	5·58	5·19, 5·97	−0·22	−0·53, 0·31	0·04	0·34	0·902	5·14	4·65, 5·63	+0·08	−0·41, 0·49	0·34	0·46	0·465

*Note.* Pre-program means are displayed to provide information about average scores in each group at baseline, while betas quantify differences between experimental groups in change scores.

*P*-values displayed are for differences between the treatment groups (PAE and EO) and the control group (reference group) in pre- to post-program changes in survey scores.

All survey questions were measured on a scale from 1 to 6 points, with higher scores indicating a higher frequency/intensity of the behaviour in question.

Estimated marginal means adjusted to reflect the influence of covariates (seasonality, age, gender, race, ethnicity, number of children, monthly food budget, distance travelled to program location, Market to MyPlate program completion and food assistance program participation) are displayed.

### Interview results

A total of eleven participants from the two treatment conditions (PAE: *n* 6, EO: *n* 5) participated in structured interviews after the conclusion of the program.

#### Program feedback

Feedback from participants about M2MP program components was largely positive, though participants also provided constructive feedback with suggestions about how to improve the program (see Table 4). Additional themes that emerged related to M2MP recipe use (as opposed to feedback) are discussed in text.

**Table 4:** Positive feedback and constructive suggestions about M2MP program components from qualitative interviews (*n* 11)

Theme	Illustrative quote
Program components which received positive feedback	
Hands-on cooking activities	One of my favourite things was actually doing it, like cutting up the vegetables. And even my daughter could cut them up, and she loved that. And it made her want to try more and stuff. So I liked that…they didn’t just cook the food and tell us to try it. We got to do it as well. That was fun. – Participant 10
Meal and budget planning	[M2MP taught] me how to plan a budget…how to plan my meals and plan within a budget…I love the program…it teaches us how to minimize [our] cost with healthy food. – Participant 7
Social aspect of program	I liked sharing meals with people, cooking with other people and working together, that was really nice. – Participant 5
Recipe books	My most favourite [part of M2MP] I think is the fact that they gave us not only the recipes, but they gave us books to be able to take home with additional recipes to be able to do at home and with our family. – Participant 1
Variety of recipes	I actually felt [the class] catered to a lot of different eating habits for people. If there were meat options, they always made sure to be inclusive of a no-meat option. That kind of catered to that group of people…The recipes were [all] different. They were not just American meals. They were kind of all over the place. But then so were the [participants]. – Participant 2
Constructive feedback to improve the program	
Customise program to participants’ needs and interests	I think one thing that should probably be included is…family planning and knowing exactly what type of families you’re working with. For me, I have nine, a household of nine *v*. someone who has a household of two. Their grocery list and mine is gonna be a lot different. I think understanding family dynamics and giving people kind of a general idea of what they need and how much they need to make a meal for, that’s equipped for their family that probably would benefit some. – Participant 2
Increase hands-on cooking	I wish we could have been a little bit more hands-on…[sometimes] it was like, “Hey we made this for you guys.” It was funner when we all were doing it together *v*.us being handed [already cooked meals] because I want to know what’s put in there, and what everything is before I eat it and stuff. – Participant 4
Increase focus on food storage techniques	Maybe if there was also along with the recipes, different methods to freeze some of the vegetables that could be frozen and kept longer. That might also be helpful. – Participant 1
Simplify meal plans and recipes taught in class	It was a lot of work to make the food, right. And there’s like the three different things that you make each [class] and I thought well if I were doing these three courses every single time for every meal, that would take me so many hours, I couldn’t do it by myself. – Participant 6

M2MP, Market to MyPlate.

There were no notable differences in feedback about the intervention between participants in the PAE and EO conditions. Hands on cooking activities, meal and budget planning and the social aspect of M2MP were the most commonly mentioned ‘favourite parts’ of the program. Participants had positive feedback about the recipe books provided, reporting that these take home resources helped them incorporate M2MP recipes at home. Additionally, participants were pleased with the variety of the M2MP recipes, noting that information about substituting ingredients based on specific dietary restrictions (e.g. meatless options) was provided, and that the program included recipes drawn from diverse cultures.

Suggestions from participants to improve M2MP included customising the program to each class of participants, increasing hands-on activities and cooking and teaching more food storage techniques. Participants suggested several different options to customise the program to each class of participants, including allowing participants to select recipes prepared in class and providing more specific guidance about meal planning based on the size of the families participating. Participants also suggested increasing hands-on activities and cooking in class. Though food storage techniques were reviewed as part of the program, participants suggested that a greater emphasis and amount of details on this information would be helpful.

While some participants said that the M2MP recipes were easy to make, others reported that they would have preferred to cook some simpler recipes in class. One participant noted that it was easier to cook recipes in class with a large group, observing that ‘there’s like ten people [in class] and everyone’s working together [so] it comes together super nicely and quickly but, trying to translate that into, you or you and two other people are doing it at home is a little tricky’ (Participant 6). It should be noted that participants only requested simpler recipes in reference to the meal plans prepared in class (not the recipe book sent home), which often involved cooking multiple recipes during the 2-h lessons.

M2MP recipes discussed in interviews included both recipes taught and cooked in class and recipes included in the M2MP recipe book that was given to participants at the end of the program. Though most participants reported using at least one of the M2MP recipes at home, a larger proportion of PAE participants (100 %, *n* 6) reported using the recipes compared to the EO participants (80 %, *n* 4). Of the ten participants who reported using M2MP recipes, seven (70 %) said they modified the recipes as needed when they used them at home. One participant explained, ‘sometimes [we don’t have] one or two [ingredients] for a recipe, then I can take another seasoning or vegetable or something to take [its] place…I think it’s like doing experiment’ (Participant 8), while another said their family enjoyed ‘mixing [the recipes] with our style and making them our own’ (Participant 3). Participants in the PAE condition also reported that the M2MP recipes helped them make use of the produce distributed during the program.

### Cooking and eating behaviours

Two broad categories of findings emerged related to cooking and eating behaviours: changes in cooking and eating attitudes and behaviours as a result of M2MP participation, and barriers and facilitators that make cooking at home more or less feasible.

#### Changes in cooking and eating behaviours

Major themes related to program impact on cooking and eating attitudes and behaviours with example quotes are provided in Table 5. All participants (100 %, *n* 11) described at least one positive change in cooking or eating attitudes or behaviours when asked how the program impacted them and their families. Participants did not describe any decreases in frequency or variety of healthy cooking or eating behaviours after participating in the program. Overall, major themes related to changes in cooking and eating behaviours were present across both treatment groups, and qualitative outcomes for PAE and EO participants were not notably different. Taken together, the key themes that emerged related to program impact suggest that participants and their families increased the variety of the foods they cooked and ate, and that this was particularly impactful for children who were reportedly more willing to try new foods after participating in the program.

**Table 5:** Reported program impact on cooking and eating behaviours of participants and their families from qualitative interviews (*n* 11)

Theme	Illustrative quote
Increased cooking self-efficacy	Trying to confidently put more vegetables together into salads has happened a lot more often [after participating in M2MP]…I feel more optimistic in general about food. – Participant 6
Increased willingness to try new foods	I think for us, actually I think for my kids, more than anything, they were more open to trying some [new foods]. – Participant 1
Trying new recipes or cooking techniques	We eat more salads nowadays, like before we [didn’t] tend to have salads…in our culture we don’t do it so much but now, we tend to make it…In our culture, we usually eat cooked vegetables but now we tend to eat raw vegetables [too]. – Participant 7
Increased variety in vegetables cooked and eaten	I’ve always incorporated a vegetable with our dinner, but now it was more like new vegetables, and how to make those. – Participant 10
Children increased participation in cooking	[M2MP] just helped me incorporate cooking with my kids and it made it funner, so anytime I cook now, we’re all in the kitchen together. – Participant 4

Participants reported increases in cooking self-efficacy, children’s willingness to try new foods, trying new recipes or cooking techniques and greater variety in the vegetables they cooked and ate after participating in M2MP. The majority of the new vegetables (such as kohlrabi, eggplant and asparagus) that participants reported eating after learning to cook them in M2MP were not dark green, red/orange, bean/pea, or starchy vegetables and instead fell in the *Dietary Guidelines for Americans* ‘other’ vegetable category^(35)^. Participants also reported introducing new dark green vegetables into their diets (e.g. broccoli) that they had not eaten prior to M2MP.

Making salads was the most common new cooking method that participants (64 %, *n* 7) reported incorporating into their meals at home. Trying a new recipe or cooking technique was more common for PAE participants (100 %, *n* 6) than participants in the EO condition (60 %, *n* 3). Overall, most participants did not increase the frequency or number of days per week that they cooked at home, but some reported that cooking became easier and less time consuming after participating in M2MP. This was especially true for participants who began to cook salads as a result of the program, since salad ingredients are often consumed raw (eliminating time needed to cook vegetables). One participant noted that, ‘[Before M2MP], [my wife] cooked three times per day. Now, she’s [still] making three meals, but…now it’s changed towards like salads…it [used to] take one and a half to two hours for cooking, but now it’s like five to ten minutes’ (Participant 3).

Participants also reported increases in their children’s involvement in cooking as a result of the program. Most notably, participants believed that their children’s participation in cooking increased their willingness to try new foods. One participant noted that ‘I found out if I’m cooking with her, she’ll try [the food we’re making]’ (Participant 10). One participant in the PAE condition also described how receiving produce allocations affected their shopping habits during M2MP, ‘when I got [produce allocations] from [M2MP], then I didn’t [need to] go to the supermarket to buy [vegetables] because I already [had them]’ (Participant 8). In addition to describing the impact of M2MP on their cooking and eating behaviours, participants also discussed barriers and facilitators to cooking at home.

#### Facilitators and barriers to cooking at home

Thematic analyses identified four factors that could act dually as both barriers and facilitators to cooking at home: meal planning and shopping, cost and budgeting, kitchen equipment and facilities and family involvement (see Table 6). Participants reported that planning and shopping ahead of time made cooking easier, while forgetting to shop or plan for meals made cooking more difficult. Some participants believed that cooking at home saved money, while others described food costs as a barrier to cooking and reported being overwhelmed trying to budget for ingredients required for home cooking. Cooking at home was more feasible if participants had the necessary kitchen equipment and space, and they described challenges presented by inadequate kitchens. While many participants said that help from family members made cooking easier, others pointed out challenges with involving children in cooking at home, since they often needed close supervision from parents. Collectively, these key themes emphasise that factors affecting home cooking can act in bidirectional ways to make it easier or harder for individuals to cook at home.

**Table 6:** Facilitators and barriers to cooking at home from qualitative interviews (*n* 11)

Theme	Illustrative quotes	
	Facilitators	Barriers
Meal planning and shopping	Are there things that make it easier for you to cook at home? Well, making a plan does…Making a plan has helped and it just takes stress off of me. – Participant 6	Most days, we try to [have vegetables] at both [lunch and dinner]. But if we didn’t shop properly, then we’re stuck. – Participant 11
Cost and budgeting	It’s definitely cheaper to [cook at home], so that’s one benefit. – Participant 5	[Budgeting] is stressful for me because it’s like I want to make a pizza but like, $15 just went into all the ingredients for my pizza and I have like $75 for my week and so this is kind of a problem. – Participant 6
Kitchen equipment and facilities	Having the necessarily utensils and all the things we need to cook makes it easier. – Participant 4	I think the biggest problem from a cooking standpoint, is that there is no ventilation system [in my kitchen]. If you want to sautéthings or you want to pan fry something, then you end up inhaling all the smoke. – Participant 11
Family involvement	Getting help makes it easier [to cook at home]…With us doing it together, teamwork, it’s better like that. – Participant 4	I know it’s not just me, and [my children] will help me [cook]. But, I still have to be there to help them help me…I think that they have the drive to want to be more involved in the kitchen. It’s just about me getting in there with them. – Participant 2

## Discussion

This embedded mixed methods study found that participation in a 7-week multi-component family-based nutrition education and hands-on cooking intervention with produce allocations was associated with improved dietary behaviours. Findings from qualitative interviews supported quantitative (survey-based) outcomes, and revealed that participants experienced increases in dietary variety, cooking self-efficacy and children’s participation in cooking after participating in the program. Interview and survey analyses allowed us to identify key factors associated with program completion (such as proximity to program location), and analyse participant suggestions to improve M2MP (such as increasing hands-on cooking and providing personalised education) that can be used to improve retention in future interventions. This research is the first cluster randomised controlled trial to examine the impact of a family-based nutrition intervention with weekly produce allocations using an embedded mixed methods design.

Participants who received produce allocations experienced significantly greater increases in fruit and vegetable consumption during M2MP than control participants. PAE outcomes did not differ from the control group for cooking frequency, or dark green and red or orange vegetable consumption. EO outcomes did not differ significantly from the control group in any behaviours measured by the survey. This finding indicates that including supplemental produce allocations can improve the effectiveness of nutrition education and cooking interventions with limited resource audiences. Though EO participants did not differ significantly from control participants in survey-based outcomes, interview findings still suggested that M2MP had a positive impact on their self-reported cooking self-efficacy, dietary variety and children’s participation in cooking. These qualitative findings add valuable information about positive impacts of M2MP beyond changes in frequency of eating or cooking behaviours (which were measured by the survey).

It is notable that reported fruit consumption increased significantly for participants in the PAE condition, despite the fact that the items distributed through the produce allocations were mostly vegetables (and not fruits). There are several possible explanations that can help us better understand the indirect effect the produce allocations had on fruit intake. Participants who received produce allocations were given resources and perhaps increased motivation that allowed them to apply what they learned in class at home, and begin to incorporate healthy cooking and eating right away during the intervention. While participants across treatment conditions reported improvements in cooking self-efficacy and attitudes towards eating a healthy balanced diet, only PAE participants were given tangible resources that allowed them to apply the knowledge gained in class immediately at home. In addition, it is possible that the vegetables provided (at no cost) to PAE participants during the program freed up money in their grocery budgets that could have been reallocated to purchase (and subsequently consume) more fruits.

Findings from thematic analyses of qualitative interview data supported the quantitative outcomes in this study and allowed for a more in-depth understanding of survey findings. Quantitative findings indicated that changes in cooking frequency did not differ significantly between participants in treatment (PAE and EO) and control conditions. This could be due in part to the high cooking frequency reported at baseline in the PAE and EO groups, which may have contributed to a possible ceiling effect. Though cooking frequency was largely unchanged (according to both survey and interview analyses), qualitative findings indicated that the variety of healthy foods cooked (and consumed) increased for participants across treatment conditions, and that cooking became quicker or easier for many after participation in M2MP.

In addition, interview findings helped explain why overall increases in vegetable intake were greater among PAE participants, but frequency of dark green and red or orange vegetable consumption did not significantly differ from the control group. Increases in overall vegetable consumption seemed to be driven by increases in consumption of vegetables that fell in the *Dietary Guidelines for Americans* ‘other’ vegetables category (consumption of ‘other’ vegetables was not assessed in the survey). Though participants did not significantly increase the amount of dark green or red and orange vegetables they reported eating (as measured by the survey), qualitative findings indicated that they did increase the variety of the vegetables they consumed within these categories. Given that participants’ frequency of consuming dark green vegetables was largely unchanged after M2MP, we can surmise that participants were likely incorporating new vegetables from the ‘other’ category to their diets without replacing or reducing their consumption of dark green vegetables.

Thematic analyses also identified several factors that could act dually as both facilitators and barriers to cooking at home, elucidating why some individuals were more or less likely to cook at home. Lastly, qualitative findings revealed key themes in positive and constructive participant feedback about M2MP components, which allowed for a better understanding of what participants liked and disliked about the program. Participants enjoyed the hands-on cooking activities, the social aspect of the program and the focus on meal and budget planning, and also appreciated the variety of recipes and the recipe book provided. Constructive suggestions to improve M2MP were also provided, including recommendations to increase hands-on cooking activities and emphasise food storage techniques, simplify meal plans and recipes and customise the program to participants’ needs and interests. This feedback could be used to inform the development of future interventions aiming to improve food behaviours in vulnerable families.

Given that there was a 33 % drop out rate, it is important to examine factors related to participant retention or program completion, which can inform recruitment and retention efforts in future interventions. Participants who lived within two miles of M2MP intervention sites were more likely to complete the program. This suggests that intervention programs implemented directly in the communities or neighbourhoods they intend to serve may be an effective approach to increasing program retention with vulnerable populations. Those with larger food budgets were also more likely to complete the program, suggesting that retention efforts should consider the challenges faced by lower resource families. Given that program completion rates varied by participant race, future interventions should make efforts to retain diverse groups of participants (such as incorporating more culturally relevant recipes) and other strategies to encourage program completion. Though childcare was provided during M2MP, participants with more children were still less likely to complete the program, indicating that future research should investigate ways to support larger families participating in similar programs and maintain participant retention.

Ko and Colleagues also conducted a mixed methods evaluation of a nutrition education program with food allocations for low-income participants^(36)^. This pre–post study found that participants increased their vegetable consumption, but unlike this study, Ko *et al.* did not detect any significant changes in fruit consumption. Similar to our study, the qualitative data collected by Ko *et al.* (via focus groups) corroborated quantitative findings and provided additional support for intervention outcomes. Smith and colleagues also implemented an intervention similar to M2MP which included two different treatment conditions (nutrition education and food allocations, and food allocations only) and a control group^(37)^. This randomised control trial targeted low-income participants and assessed carotenoid scores, a physiological measure of nutrients that serves as a marker for consumption of certain fruits and vegetables (e.g. sweet potatoes, carrots, tomatoes, mangos) which were distributed during the intervention. Smith *et al.* found that participants in the education and food allocations treatment experienced significantly larger increases in carotenoid scores compared to participants who only received food allocations and the control group. Taken together, the findings from the present study and from Smith *et al.* indicate that multicomponent interventions addressing environmental aspects, such as food access, that provide both education and increased access to healthy foods may be an effective way to support improvements in dietary behaviours in low resource families.

Qualitative findings in this study identified several bidirectional factors that could act as both barriers and facilitators to cooking at home. Several of the key themes discovered in this study, including the impact of kitchen equipment or facilities, budgeting and affordability and organisation and meal planning have been identified in past research^(19,38,39)^. One novel theme identified in this study is the dual influence that assistance from family members (specifically children) can have on cooking. We found that help from family members can make it either harder or easier to cook at home based on their level of competence, independence and cooking skills.

This study is not without limitations. Though the study design allowed us to examine changes from pre- to post-intervention in targeted outcomes, there was no longitudinal follow up with participants to assess the long-term impact of M2MP. We were also unable to conduct interviews with participants who did not complete the program, and therefore could not probe further to identify factors that led them to drop out of M2MP. Given that this was an exploratory study, findings should be considered preliminary and should be confirmed and replicated in future research. All interventions took place in central Illinois, and findings may or may not be generalisable to broader populations. Future research should assess the efficacy of nutrition interventions with produce allocations across more geographically diverse populations. Despite these limitations, this study also had a number of strengths and makes and important contribution to the literature.

This study used a rigorous mixed methods design with two different treatment conditions to better understand the impact that produce allocations had (above and beyond standard nutrition and cooking education) on participant outcomes. M2MP was also unique in that it built on social factors allowing the whole family to participate, and did not only target the main meal preparer. In addition, this program emphasised hands-on cooking experiences (not just demonstrations or tastings) that allowed participants to actively practice the cooking skills learned in class. Lastly, this program addressed community and environmental factors by teaching a diverse group of limited resource participants about local resources (e.g. farmers’ markets) that could help them purchase and consume more fresh fruits and vegetables.

## Conclusions

In this study, participants in the PAE condition who received weekly allocations which directly increased access to fresh produce reported significant improvements in fruit and vegetable consumption (compared to the control group). Though the findings of this study are modest, the study has several notable strengths that make a unique contribution to the literature about nutrition interventions with low resource populations. The results demonstrate that interventions that use a multi-component social ecological design can have greater impact on low-income families that face numerous challenges in consuming healthful food and improving diet quality.

## Supplementary material

For supplementary material accompanying this paper visit https://doi.org/10.1017/S1368980021003797. click here to view supplementary material
